# El Niño drives a widespread ulcerative skin disease outbreak in Galapagos marine fishes

**DOI:** 10.1038/s41598-018-34929-z

**Published:** 2018-11-09

**Authors:** Robert W. Lamb, Franz Smith, Anaide W. Aued, Pelayo Salinas-de-León, Jenifer Suarez, Marta Gomez-Chiarri, Roxanna Smolowitz, Cem Giray, Jon D. Witman

**Affiliations:** 1 Brown University, Department of Ecology and Evolutionary Biology, Providence, RI 02912 USA; 2 Universidade Federal de Santa Catarina, Departamento de Ecologia e Zoologia, Florianopolis, Brazil; 3 Department of Marine Sciences, Charles Darwin Research Station, Av. Charles Darwin s/n, Puerto Ayora, Galapagos Islands Ecuador; 4 Pristine Seas, National Geographic Society, Washington, D.C. USA; 5 Parque Nacional Galápagos, Puerto Ayora, Ecuador; 6 University of Rhode Island, Narragansett, RI USA; 7 Roger Williams University, Bristol, RI USA; 8 Kennebec River Biosciences, Richmond, ME USA

## Abstract

Climate change increases local climatic variation and unpredictability, which can alter ecological interactions and trigger wildlife disease outbreaks. Here we describe an unprecedented multi-species outbreak of wild fish disease driven by a climate perturbation. The 2015–16 El Niño generated a +2.5 °C sea surface temperature anomaly in the Galapagos Islands lasting six months. This coincided with a novel ulcerative skin disease affecting 18 teleost species from 13 different families. Disease signs included scale loss and hemorrhagic ulcerated patches of skin, fin deterioration, lethargy, and erratic behavior. A bacterial culture isolated from skin lesions of two of the affected fish species was identified by sequencing of the 16S rRNA gene as a *Rahnella* spp. Disease prevalence rates were linearly correlated with density in three fish species. In January 2016, disease prevalence reached 51.1% in the ring-tailed damselfish *Stegastes beebei* (n = 570) and 18.7% in the king angelfish *Holacanthus passer* (n = 318), corresponding to 78% and 86% decreases in their populations relative to a 4.5-year baseline, respectively. We hypothesize that this outbreak was precipitated by the persistent warm temperatures and lack of planktonic productivity that characterize extreme El Niño events, which are predicted to increase in frequency with global warming.

## Introduction

As one of the most pervasive anthropogenic impacts on the planet, climate change affects a myriad of physical processes that govern the conditions for life^1^. In marine ecosystems, increased greenhouse gases are altering both ocean chemistry and circulation^2,3^, which can cause abrupt and persistent changes in local sea surface temperature (SST) and productivity^4^. The combination of enhanced radiative forcing and altered circulation can also exacerbate natural phenomena such as the El Niño-Southern Oscillation (ENSO), increasing the frequency of extreme climate events^5,6^. The three most extreme El Niño events on record all occurred in the past four decades and have been progressively stronger^7^, suggesting that the frequency and magnitude of major ENSO events will increase with global warming^5,6^.

Extreme events such as El Niño pose one of the largest global threats to ocean ecosystem health^2^, and there is growing concern regarding how climate change will foster the spread of disease^8,9^. Diseases of marine organisms have been extensively studied in high-value organisms such as oysters^10^, and are particularly prevalent in aquaculture conditions^11^, where high densities and stagnant water tend to favor outbreaks and where it is easier to detect, track, and manage disease. Global warming has accelerated the reporting of disease in wild marine populations by pushing organisms such as corals past their thermal tolerance limits, resulting in coral bleaching and increased susceptibility to associated pathogens^12,13^. Considerable focus has also been given to human health concerns such as increased incidence of harmful algal blooms with warming ocean temperatures^14^. Less is known regarding the etiology and implications of disease in wild finfish populations^15^, or how these will be affected by climate change^16^.

Most disease outbreaks reported from wild fish populations involve multiple pathogens and are associated with concomitant environmental stress^15–18^. In the Galapagos Islands, El Niño periods are characterized by increased SST and decreased marine biomass production^19,20^. Higher temperatures can facilitate pathogen virulence, survival, and transmission rates^9^, while lower productivity can depress host population parameters and individual body condition, thus compromising fish immune response^21,22^. Major El Niño events (1982–83, 1997–98) during which the SST anomaly reached a sustained value of at least +2 °C were associated with reduced fish biomass, live coral cover, algal and planktonic productivity, and top predator abundances^19^. However, the lack of replicated monitoring across these strong climate perturbations severely limits the scope of mechanistic inference as to the specific effects of El Niño on fish communities. Here, we report on an outbreak of a novel wild fish disease using a 4.5-year study of marine communities on rocky reefs of the Galapagos Islands before, during, and after the extreme El Niño of 2015–16. The disease appeared at the peak of the El Niño event and affected 18 different species across 6137 km^2^ of the archipelago and was associated with up to 86% decreases in affected fish populations. To our knowledge this is the first study to evaluate the impact of warming oceans on a disease outbreak in wild finfish and can serve as an indicator of the potential devastating impacts of climate change on wild fish populations.

## Results and Discussion

### Physical oceanography

**Figure 1:**
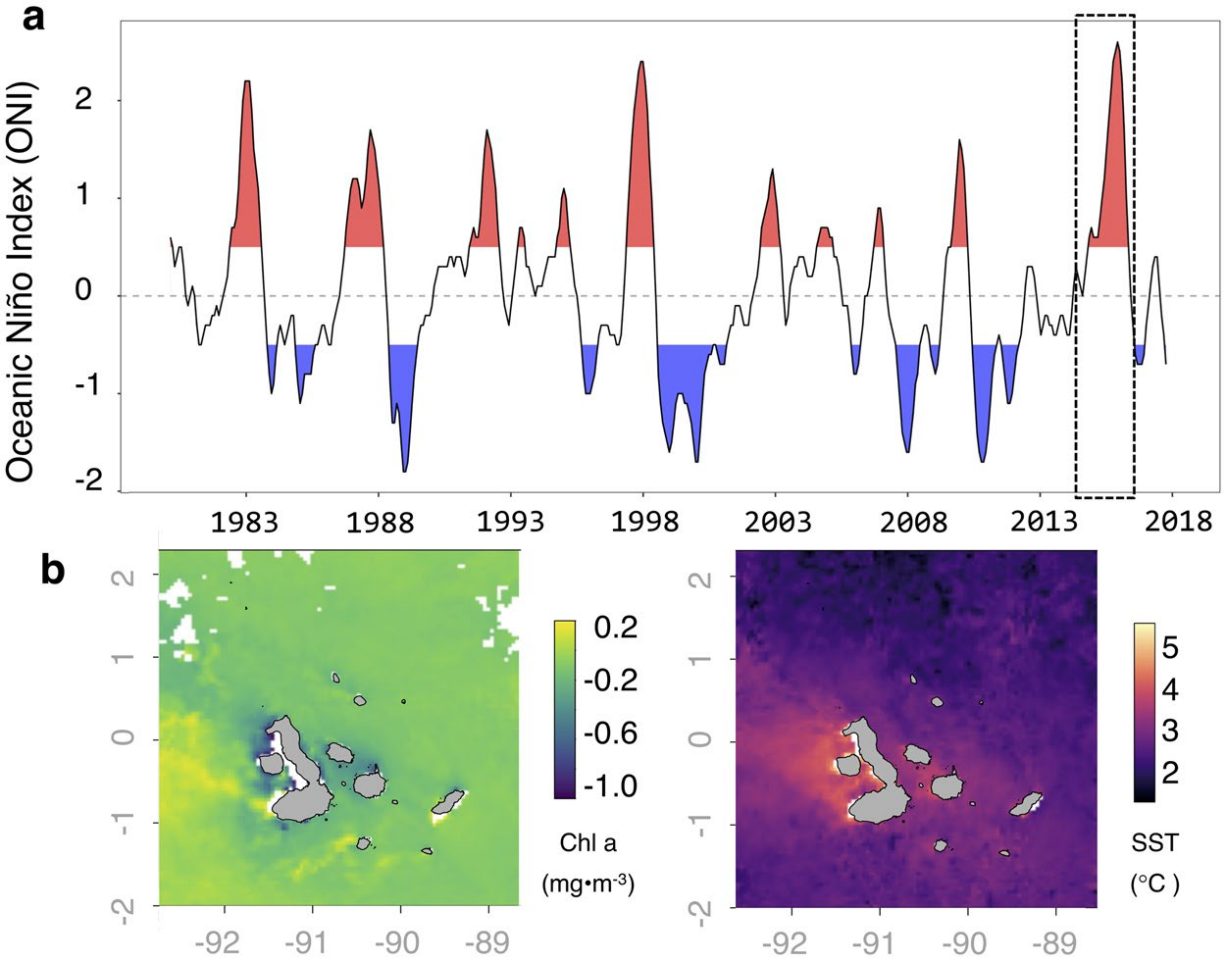
Physical oceanography characterizing the extreme El Niño event of 2015–16. (**a**) 2015–16 was the strongest El Niño since the extreme events of 1982 and 1997, based on Oceanic Niño Index (ONI). El Niño events are depicted in red, La Niña events are in blue. (**b**) Anomalies for sea surface chlorophyll *a* in mg/m^3^ and (**c**) sea surface temperature in °C relative to a 14-year mean in the Galapagos Islands region (grey x and y-axes denote longitude and latitude) during the peak of the El Niño event (baseline: Jan 13, 2003–Nov 21, 2017; El Niño period: Dec 29, 2015–Jan 29, 2016; dashed box in panel a).

The Galapagos Islands are situated in the Tropical Eastern Pacific and are very sensitive to ENSO oscillations^20^. The equatorial undercurrent (EUC) supplies the archipelago with cool, nutrient-rich waters that make the Galapagos uniquely productive for sub-tropical seas^23^. However, during El Niño years, the trade winds that drive equatorial upwelling and the EUC slacken, producing warm SST anomalies. This in turn stratifies the surface ocean, pushing the thermocline between deep cold water and warm surface water below 60 m depth and limiting marine productivity in the photic zone. The 2015–16 El Niño event reached an Oceanic Niño Index (ONI) value of 2.6, joining and surpassing the events from 1997 and 1982 as the three strongest El Niño events in the ONI record^7^ (Fig. 1a). Satellite measurements revealed negative sea surface chlorophyll *a* anomalies within our study area ranging between −0.2 and −0.8 mg/m^3^ that persisted between September 2015 and April 2016 (Fig. 1b). This coincided with a maximum SST of 30.0 °C, and an archipelago-wide positive SST anomaly of +2.5–4.0 °C during the same period (Fig. 1c).

### Fish surveys

**Figure 2:**
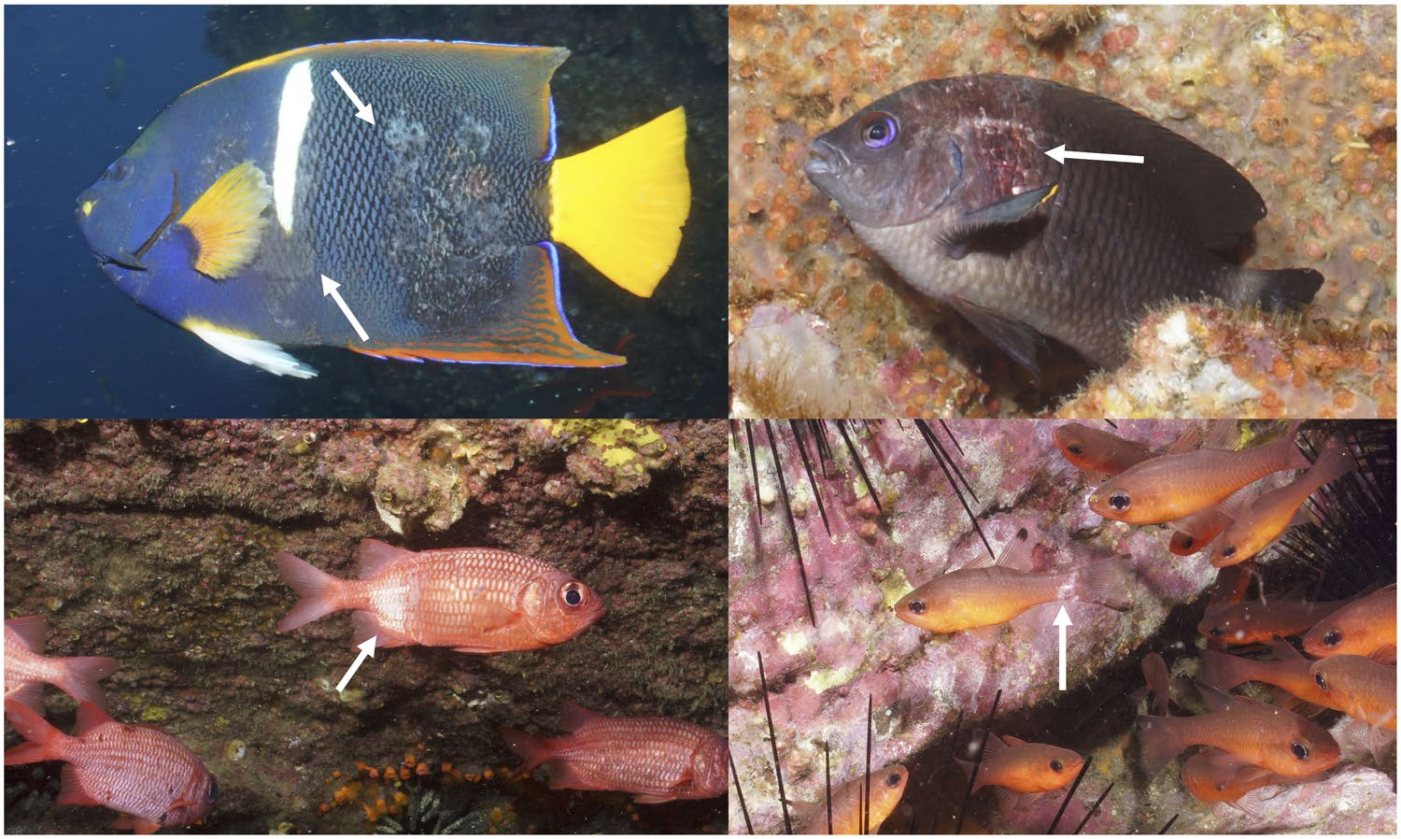
Composite photograph of four of the fish species observed with ulcerative skin disease at the height of the El Niño event in January 2016. The characteristic lesions are indicated by white arrows, manifesting as circular white patches on the skin or larger areas of scale dehiscence and eroding skin that show sub-epidermal hemorrhaging tissue. Clockwise from top left: *Holacanthus passer, Stegastes beebei, Apogon atradorsatus*, and *Myripristis leiognathus*. Photo credits: J. Witman. Also see Supplementary Video 1 of diseased fish.

On January 8, 2016, at the height of the El Niño event, we discovered the outbreak of a novel ulcerative skin disease in wild fish populations while diving at the island of Cuatro Hermanos East (Table 1), as part of a long-term ecological monitoring effort^24^. We observed the following spectrum of disease signs that appeared to be at different stages of disease progression: from small (0.5–1 cm diameter) multifocal white patches of skin on the sides, head, and fins, to larger areas (>2 cm diameter) of scale loss and exposure of necrotic, eroding skin to reveal hemorrhaging sub-epithelial tissue (Fig. 2, Supplementary Fig. S1), culminating in advanced cases in which >50% of the skin was covered in ulcerative lesions. Heavily affected fish had reduced functionality of important structures, including deteriorated fins that appeared torn and ragged, ulcers around the mouth, and subacute keratitis and erosion of the cornea. We also observed aberrant behavior including rubbing the surface of the body against rocks, lethargy, and increased gill ventilation rates (Supplementary Video S1). These disease signs were superficially similar to scale-drop disease, known from the mariculture of barramundi *Lates calcifer* in southeast Asia^11^, but an agent-specific real time PCR assay for the virus that causes scale-drop disease was negative for our samples. Another similar ulcerative skin disease has been reported in California, USA, but was restricted to a single damselfish species^25^. Ulcerative skin diseases are a relatively common occurrence at low prevalence rates in wild fish populations (Supplementary Table S1), but large outbreaks are almost always associated with environmental triggers such as pollution or toxins that produce skin damage, which is subsequently colonized by environmental bacteria^15,26^. Ulcerative skin diseases are thus generally referred to as a syndrome with multiple pathogenic agents. For instance, a fin rot disease reported from the New York Bight affected 22 different species of wild fishes^17^, but that outbreak was attributed to proliferation of *Vibrio, Aeromonas*, and *Pseudomonas* bacteria due to severe industrial pollution and human-driven coastal eutrophication. In contrast, the Galapagos Islands are extremely isolated and oceanic, with strict controls on human population and impacts, indicating that ulcerative skin disease outbreaks can also occur in relatively pristine ocean ecosystems.

**Table 1:** List of first sightings of diseased species (n = 18) by date and site along with species trophic category and taxonomic family.

Site	Day (Jan 2016)	Species	Common name	Family	Trophic category
Cuatro Hermanos East	08	*Holacanthus passer*	King angelfish	Pomacanthidae	Sessile invertivore
Cuatro Hermanos East	08	*Myripristis leiognathus*	Panamic soldierfish	Holocentridae	Planktivore
Cuatro Hermanos East	08	Stegastes beebei*	Ring-tailed damselfish	Pomacentridae	Omnivore
Caamaño	12	*Microspathodon dorsalis*	Giant damselfish	Pomacentridae	Mobile invertivore
Cuatro Hermanos GW	13	Apogon atradorsatus*	Blacktip cardinalfish	Apogonidae	Planktivore
Cuatro Hermanos East	13	*Bodianus diplotaenia*	Streamer hogfish	Labridae	Mobile invertivore
Cuatro Hermanos East	13	*Chromis atrilobata*	Brown chromis	Pomacentridae	Planktivore
Cuatro Hermanos East	13	*Diodon holocanthus*	Balloonfish	Diodontidae	Mobile invertivore
Cuatro Hermanos East	13	*Epinephelus labriformis*	Flag cabrilla	Epinephelidae	Piscivore
Cuatro Hermanos GW	15	Gobioclinus dendriticus*	Bravo clinid	Labrisomidae	Mobile invertivore
Cuatro Hermanos GW	15	Ophioblennius steindachneri*	Panamic fanged blenny	Blenniidae	Omnivore
Rocas Beagle	18	Stegastes arcifrons*	Yellowtail damselfish	Pomacentridae	Omnivore
Roca Cousins	19	*Muraena lentiginosa*	Jewel moray	Muraenidae	Piscivore
Roca Cousins	19	Orthopristis forbesi*	Galapagos grunt	Haemulidae	Mobile invertivore
Roca Cousins	19	*Paranthias colonus*	Pacific creolefish	Epinephelidae	Planktivore
Rocas Gordon	21	*Acanthurus xanthopterus*	Yellowfin surgeonfish	Acanthuridae	Omnivore
Rocas Gordon	21	Prionurus laticlavius*	Razor surgeonfish	Acanthuridae	Omnivore
Caamaño	23	Pareques perissa*	Galapagos drum	Sciaenidae	Mobile invertivore

*Eastern Tropical Pacific (ETP) regional endemic.

We observed 18 out of a total richness of 93 fish species (19.4%) showing signs of ulcerative skin disease across all study sites (Table 1). Notably, there were no unifying taxonomic or trophic traits across all affected species that might point to a common source of infection: teleosts of 13 different families and five different trophic groups were represented, many of which are regional endemics to the Eastern Tropical Pacific bioregion. Underwater observations on SCUBA revealed a common ecological trait among affected species, in that all are susceptible to common skin abrasion and damage. Damselfishes such as *Stegastes beebei, S. arcifrons*, and *Microspathodon dorsalis* are extremely territorial and are constantly biting and harassing neighbors to enforce their territories^27^. Another group of species commonly suffers attacks by parasitic false cleaner fish, the sabertooth blenny *Plagiotremus azalea* (R.W.L, personal observation), which takes chunks of skin and mucus from unsuspecting client fish (e.g., *Holacanthus passer*, *Apogon atradorsatus, Paranthias colonus*, and *Prionurus laticlavius*). Still other species are often observed carrying high ectoparasite loads (*Myripristis leiognathus*). All of these interactions generate small wounds and abrasions in the skin that are potential entryways for environmental pathogens.

**Figure 3:**
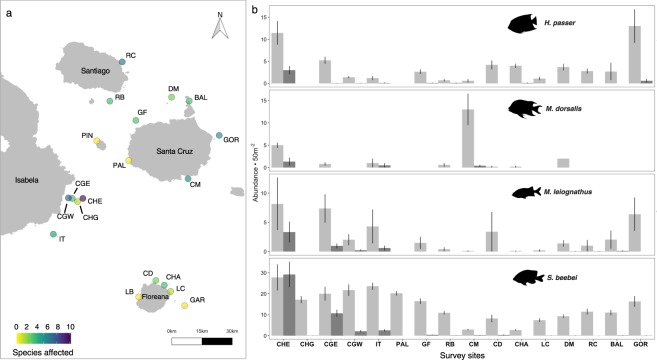
Distribution of ulcerative skin disease among sites and within populations of fish species across 6137 km^2^ of the central Galapagos Marine Reserve. (**a**) Map of the central Galapagos Islands indicating all sites where surveys took place (n = 19) and the number of species observed with lesions at each site. See Supplementary Table 2 for site details. (**b**) Mean and standard error of density per 50 m^2^ of healthy (light grey bars) and diseased (dark grey bars) fish at 16 sites where targeted transects were carried out for the four most heavily infected species during the month of January 2016. Fish illustrations provided by David Buckner.

The outbreak was widely distributed throughout the survey area and was present at 15 of 19 sites encompassing 6137 km^2^ of the central archipelago (Fig. 3a and Supplementary Table S2). However, patterns of high prevalence were clearly spatially segregated, with the highest number of species affected and highest prevalence across all species observed within the Cuatro Hermanos group of islands off the southeastern coast of Isabela Island. Crevices in the reef are particularly abundant at the Cuatro Hermanos East Island, where we often observed several species of fish with lesions, especially *M*. *leiognathus*, coming into physical contact with each other and potentially increasing disease transmission rates. Cuatro Hermanos East is also home to one of the largest and most heavily visited fish cleaning stations in our study region (R.W.L., personal observation). This cleaning station is dominated by barberfish *Johnrandallia nigrirostris*, which may act as disease vectors. During the outbreak, we observed several instances of affected *M. leiognathus*, a normally cryptic, nocturnal fish that is never seen receiving cleaning, visiting the *J. nigrirostris* station in full light over the open reef and aggressively soliciting cleaning of ulcerated skin (Supplementary Video S2).

Ulcerative skin disease was notably absent from La Botella and Gardner sites around Floreana Island and Las Palmas and Pinzón sites to the southwest of Santa Cruz Island (Fig. 3a). These four sites were unique in that they experienced only 4 weeks during which temperatures surpassed 26 °C between September 2015 and January 2016, whereas SST at other sites remained above 26 °C for 6–8 weeks. As such, there was a positive linear relationship between the number of weeks of SST above 26 °C and the log(x + 1) total number of species affected by ulcerative skin disease across sites (*P* = 0.00123, R^2^ = 0.505; Supplementary Fig. S2). This suggests that the appearance of ulcerative skin disease is positively temperature dependent.

**Figure 4:**
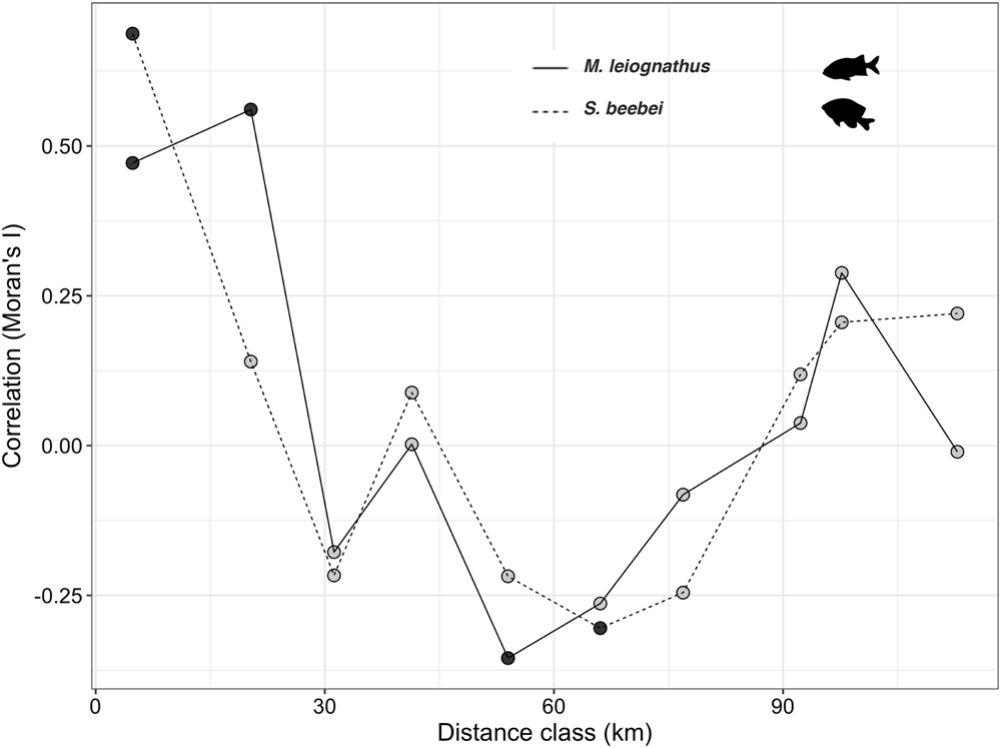
Analysis of spatial auto-correlation of disease prevalence across sites. The correlogram depicts values of Moran’s I correlation coefficient, with positive values indicating positive spatial auto-correlation at that distance class and negative values indicating negative auto-correlation. Significant values (*P* < 0.05) are indicated by solid circles, and distance classes are connected by a solid line (*M. leiognathus*) or dashed line (*S. beebei*). Fish illustrations provided by David Buckner.

**Figure 5:**
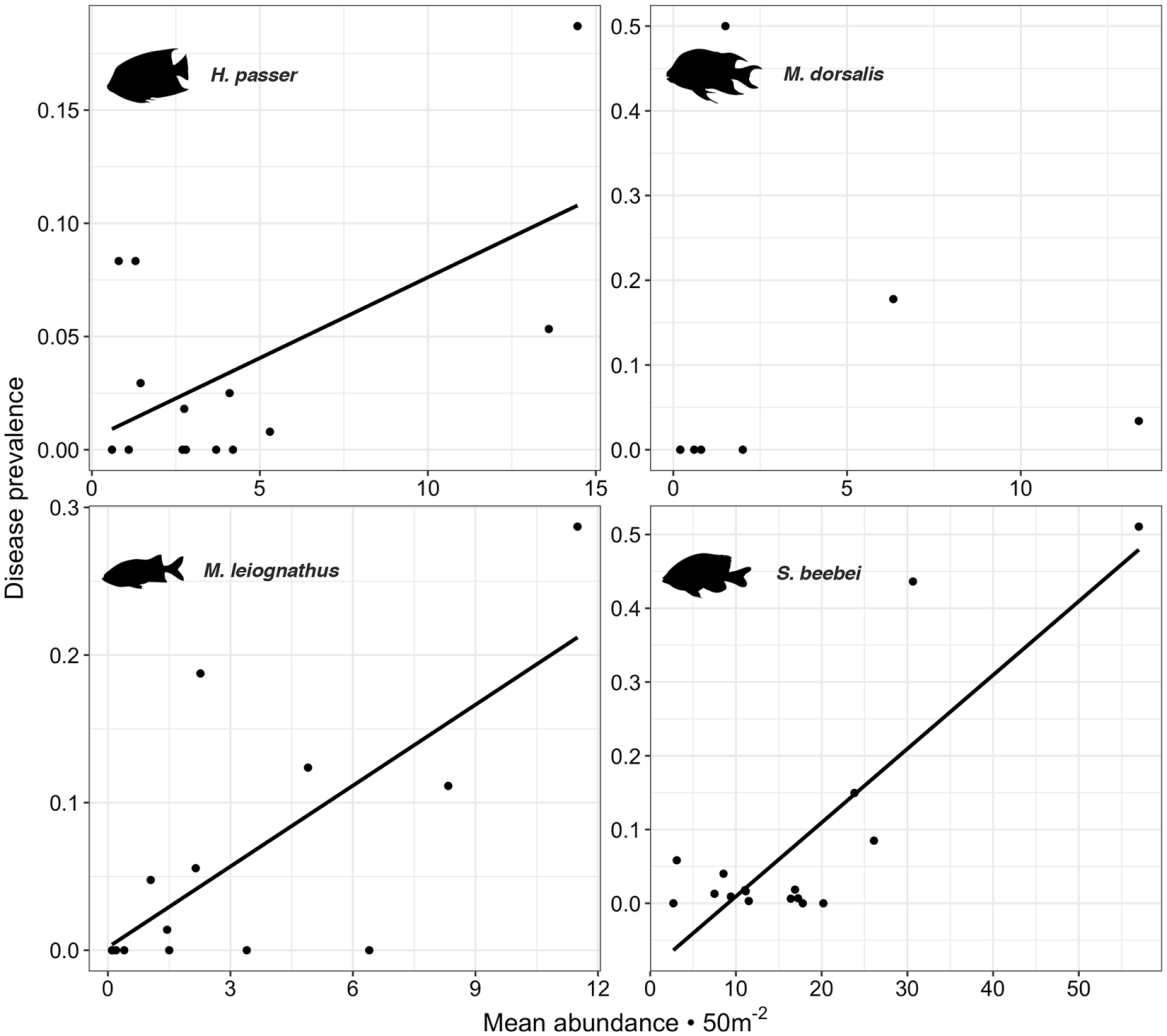
Density-dependence of fish disease prevalence. Linear regression analysis revealed that the prevalence of ulcerative skin disease (proportion of total number of individuals exhibiting disease signs) for *S. beebei, H. passer*, and *M. leiognathus* exhibited density dependence across all survey sites. *M. dorsalis* did not exhibit this phenomenon. Regression equations are: *S. beebei:* y = 0.01x − 0.091, *P* < 0.001, R^2^ = 0.683; *H. passer:* y = 0.007x + 0.005, *P* = 0.026, R^2^ = 0.294; *M. leiognathus:* y = 0.018x + 0.002, *P* = 0.004, R^2^ = 0.473; and *M. dorsalis:* y = 0.0007x + 0.087, *P* = 0.966, R^2^ = −0.166. Note different scales for x and y axes for the different species. Fish illustrations provided by David Buckner.

Of the 18 species observed with signs of disease, four “targeted species” were censused intensively due to prevalence rates surpassing 15% of the population in at least one site (Fig. 3b). Peak disease prevalence was observed at Cuatro Hermanos East for all species: *H. passer*: 18.7% (n = 318)*, M. dorsalis*: 17.7% (n = 38), *M*. *leiognathus*: 28.7% (n = 138), and *S*. *beebei*: 51.1% (n = 570). Disease prevalence was spatially clustered across sites for both *S. beebei* and *M. leiognathus* (but not for *H. passer* or *M. dorsalis*), with positive spatial auto-correlation at near distances classes <20 km, and negative auto-correlation at intermediate distance classes of 55–70 km (Fig. 4). This pattern was driven by the cluster of high prevalence at sites within the Cuatro Hermanos group of islands, which is 50–70 km from the sites of very low prevalence around Floreana Island. A similar negative correlation at the 30 km distance class, though non-significant, corresponded to low prevalence sites around the southwestern corner of Santa Cruz Island. There was also a significant linear relationship between fish abundance and disease prevalence at each site for *S. beebei* (*P* < 0.001, R^2^ = 0.683)*, H. passer* (*P* = 0.026, R^2^ = 0.294), and *M*. *leiognathus* (*P* = 0.004, R^2^ = 0.473), but not for *M*. *dorsalis* (*P* = 0.966, R^2^ = −0.166). This suggests that the prevalence of ulcerative skin disease is positively density dependent (Fig. 5).

**Figure 6:**
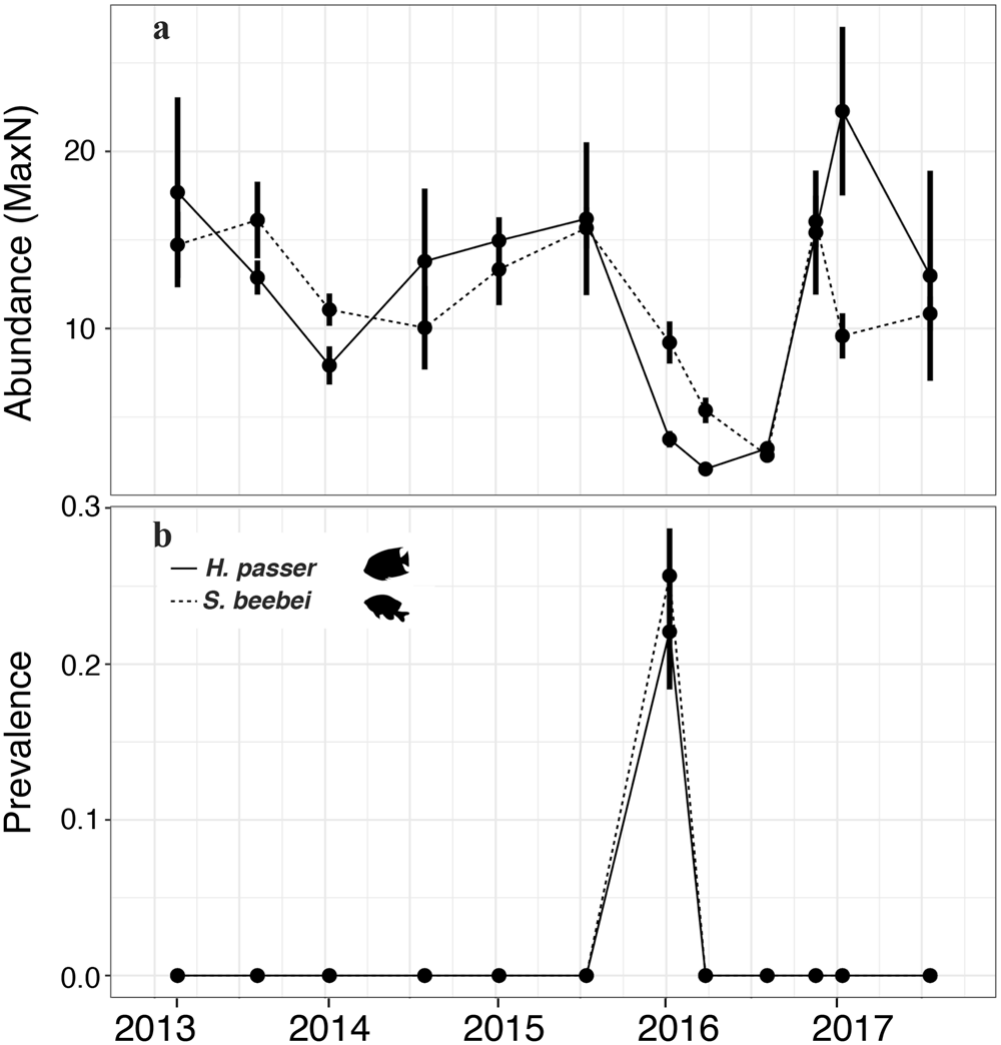
Disease prevalence and abundance over time of *H. passer* and *S. beebei* at Cuatro Hermanos East. We analyzed eight 15-minute stationary videos every six months from Cuatro Hermanos East Island for the period of January 2013–July 2017 to estimate abundance (**a**) and disease prevalence (percentage of total number of individuals exhibiting disease signs) (**b**) for *Holacanthus passer* (solid line) and *Stegastes beebei* (dashed line). Data shown are mean and standard error, and abundance is quantified as the maximum number of individuals observed in a single frame (MaxN) standardized to field of view. Fish illustrations provided by David Buckner.

Bi-annual video monitoring of *S. beebei* and *H. passer* populations from 2013–2017 revealed a clear link between the timing of the peak of the 2015–16 El Niño event and the prevalence of ulcerative skin disease. The disease was completely absent from the community at Cuatro Hermanos East until January 2016, and prevalence decreased precipitously once El Niño conditions subsided (Fig. 6). Prevalence rates were below 5% by March 2016, and signs of the disease had disappeared completely by August 2016. It has not been observed in bi-annual surveys since. We also reviewed footage from a single stationary video camera in 2003 from Cuatro Hermanos East and observed no sign of lesions consistent with ulcerative skin disease. Furthermore, no such disease signs have ever been reported despite a continuous presence of research divers from Brown University, the Galapagos National Park, and the Charles Darwin Foundation throughout the archipelago since at least 1999. Indeed, it is only due to a regular bi-annual monitoring program^24^ carried out by the authors during this same period that ulcerative skin disease was first discovered, underscoring the need for long-term monitoring to detect marine disease outbreaks and ecological change in response to anthropogenic impacts^28^.

Analysis of fish abundance from remote videos deployed bi-annually over the 4.5-year period revealed a mortality event coincident with the outbreak that departed significantly from background population fluctuations in both *H. passer* and *S. beebei* at Cuatro Hermanos East (Fig. 6a). In August 2016, the mean abundance of *S. beebei* at this site decreased significantly from a baseline of 12.67 individuals to 2.83 individuals, a 78% decrease (maximum number of individuals in a single frame -MaxN- per 15 minutes of video analyzed; one-way ANOVA: *F* = 8.4, *P* < 0.001, Supplementary Table S3). *S. beebei* is an important ecosystem engineer since it protects algal territories from hyper-abundant and voracious herbivorous fishes and urchins^27^, thus increasing algal productivity. The 78% reduction in *S. beebei* populations during the disease outbreak at Cuatro Hermanos East likely impaired this important ecosystem function. Another damselfish, the endemic Galapagos blackspot chromis *Azurina eupalama*, has not been observed since the 1982/83 El Niño despite intensive observations and is believed to be extinct^29–31^. The demise of *A. eupalama* was attributed to its dependence upon planktonic food, which became scarce during El Niño, but our findings suggest that a disease outbreak could also have been implicated in its decline.

The abundance of *H. passer* similarly decreased over the same period by 86% from an average MaxN of 14.83 to 2.07 individuals (MaxN; *F* = 9.7, *P* < 0.001, Fig. 6a, Supplementary Table S3). In addition to the ulcerative skin disease outbreak, several other factors associated with the extreme El Niño event may have contributed to mortality in both species, especially considering that the population decreases were considerably greater than prevalence rates observed in the field. For instance, mortality may have been exacerbated by the general lack of planktonic food as a result of relaxed upwelling^20^. The anomalously high temperatures may also have directly caused fish mortality or caused fish populations to migrate seeking a cooler refuge. This is less likely for *S. beebei*, which is a territorial demersal species. In addition, we did not observe dead fish in the water, although the high densities of large piscivores in the Galapagos may result in terminally ill reef fish quickly being captured by predators^32,33^. Dead fish may also be flushed offshore by strong currents, which are common at many of the study sites^24^. We did observe extremely lethargic and disoriented individuals in advanced stages of disease, especially of *S. beebei* and *M. leiognathus*, which were not seen again on subsequent censuses 1–2 weeks later. Regardless of the contribution of direct thermal stress, migration, disease, or other unknown factors to population decreases during El Niño in *S. beebei* and *H. passer*, rapid recovery of both species in November 2016 corresponded with a strong La Niña period of cold, nutrient-rich water conditions^7^, which may serve as a mechanism of resilience following El Niño-associated mortality.

### Pathogen isolation and identification

The tissues of four species of affected fish were examined for microscopic lesions and the presence of potential pathogens. Internal organs showed no signs of disease. Skin tissues selected for histological examination were sampled from the edges of the erosion/ulcers or from the centers of the ulcers. The microscopic appearance of the lesions was similar between species. The skin of all diseased fish showed multifocal areas of dermatitis characterized by ulcerations and erosions of the epithelium, intra- and inter-epithelial and dermal edema, mixed inflammatory cell infiltration in the dermis and epithelium, vascular congestion, and focal hemorrhage (Supplementary Fig. S3). Areas at the edges of the ulcers showed epithelial erosion with dermal congestion, edema, vascular congestion, and mixed inflammatory cell accumulation. In some areas rafts of dead and dying epithelial cells were sloughed from the underlying dermis (degloving). In areas of epithelial necrosis, occasional eosinophilic to amphiphilic inclusions were noted in the nuclei or cytoplasm of the epithelial cells and were thought to result from the degeneration of the epithelial cells. Aggregations of rod-shaped bacteria were noted in areas of ulceration in 5/24 fish examined, and rarely in the scale pockets in affected areas. However, bacteria overlying and loosely attached to the surface of ulcerated tissue are often washed off fish skin during histological processing, so more bacteria may have been present in live tissues. There were also no signs of parasites or fungal infections in tissue analyses.

Two *S. beebei* and one *M. leiognathus* were kept frozen for pathogen screening. All three fish showed signs of skin lesions with light hemorrhaging between scales and epidermis. In addition to scales readily sloughing off the skin, in regions near the dorsal fin where the lesions appeared more advanced, the skin was observed to be separating from the muscle layer underneath. Bacterial growth was not observed from brain and kidney tissue samples on any of the media and incubation temperatures utilized. Skin lesion samples from one of the *S. beebei* and the *M. leiognathus* produced a pure growth of a beta hemolytic, Gram negative, facultatively anaerobic organism in low abundance. Further biochemical testing using API-20E (bioMerieux Inc.) kits preliminarily identified the bacterium as a member of the genus *Pantoea* in the family Enterobacteriaceae. Additional evaluation using 16S rRNA gene sequencing showed close identity to sequences in GenBank for only two organisms [GenBank accession number MK108079], also belonging to Enterobacteriaceae. The closest identity (99.3%, 1407/1417 bp) was to *Rahnella inusitata*, a species recently described from terrestrial sources^34^. *Rahnella* species have been described from the rhizosphere, phyllosphere, seeds, fruits, water and intestinal tracts of herbivores including invertebrates. Some *Rahnella* species, such as *R. aquatilis* found in freshwater and soil, have been associated with infections in humans, usually immunocompromised and trauma patients^35,36^. The next closest identity (98.7%, 1398/1417 bp) was to *Rouxiella chamberiensis*, which was described from a case involving infant mortalities due to contaminated parenteral nutrition bags^37^. Sequence identity to other bacterial organisms was significantly lower, at 98% and below and mainly to *Hafnia* and *Serratia* species. While *Hafnia* and *Serratia* species are commonly isolated from finfish, sometimes being involved in opportunistic infections, reports of isolation of *Rahnella* and *Rouxiella* species mainly consist of *R. aquatilis*^38^. Although presence of opportunistic agents (e.g. *Aeromonas, Pseudomonas* or *Shewanella sp*.) or organisms associated with skin lesions in other marine species (e.g. *Tenacibaculum* or *Vibrio sp*.) were expected, none of these were detected. This could potentially be due to the sampling of leading edges of lesions and avoidance of central regions of ulcers, which could potentially contain a greater abundance of secondary organisms. The detection of a rare organism, rather than those which are more abundant in the environment, suggests that sample contamination during or after collection is not likely to be the source. However, the low level of detection in bacterial culture from two out of three fish sampled does not provide sufficient confidence that this bacterium was the causative agent. The frozen nature of the specimens before sampling also could have affected the results. Challenge experiments would need to be performed to determine the ability of the bacterial isolate from diseased fish to cause disease.

No cytopathic effect was detected in live viral culture assays using multiple fish cell lines. Screening for scale-drop disease virus using real-time PCR was also negative. We cannot fully rule out the involvement of a viral pathogen since some fish viruses cannot be isolated in conventional cell lines. Future studies using electron microscopy would help confirm our negative virology results.

It is unclear why this disease has never before been reported in the Galapagos, as many diseases are common at low prevalence rates in wild fish populations under normal conditions^8^. The causative agent may have been introduced from source areas via maritime shipping^39^ or by altered current patterns, which are known to distribute tropical marine species into the colder sub-tropical areas of the region during El Niño events^40^. El Niño conditions could also generate changes in water chemistry or the community composition of plankton, releasing toxins into the water column^18^. There is also evidence that small human settlements on oceanic islands such as those in the Galapagos can alter the composition of marine microbial communities^41^. Artisanal fishing within the multi-use Galapagos Marine Reserve has greatly reduced the abundance of lobsters and sea cucumbers^42^, which are important bottom-feeding invertebrates and detritivores whose absence may have further exacerbated the risk of disease outbreaks during the recent ENSO period.

## Conclusions

We have described the first known case of widespread ulcerative skin disease in reef fishes of the Galapagos Islands. The clear association between this disease outbreak and the recent extreme El Niño event strongly suggests a regional climatological trigger that generated thermal and nutritional stress and facilitated pathogenicity by microorganisms present in the ecosystem. This temperature dependence is supported by the fact that there were some sites where waters remained cool enough to prevent outbreaks (Floreana Island, SW of Santa Cruz Island). Although we cannot determine causality directly, it is likely that the persistent high temperatures, low productivity, and altered current patterns driven by El Niño were involved. This inference is strengthened by the absence of such outbreaks described in the past, concurrent disease signs in 18 different teleost species, and the disappearance of the disease with the cessation of the extreme climate event. Multi-species outbreaks of ulcerative skin disease are often driven by initial skin damage resulting from environmental stressors, such as crowding in aquaculture, pollution, and extreme temperature perturbation, which is then colonized by multiple environmental pathogens^15,26^. Since the pathogenicity of the bacterial strain isolated from diseased fish in the Galapagos has not been tested, it is inconclusive whether the outbreak was caused by an opportunistic pathogen colonizing pre-existing wounds, or a primary pathogen capable of causing infection under these environmental conditions. Regardless, the 18 affected species of fish had density-dependent disease prevalence at sites where warm SST anomalies prevailed. This general epidemiology may be favored by future extreme El Niño events and climate change in general^1,6^.

The relatively pristine waters of the Galapagos Islands imply that ulcerative skin disease may become common in wild fish populations even in the absence of pollution and coastal eutrophication. It is difficult to mitigate the local effects of large-scale stressors such as diseases driven by climate change and ENSO cycles. Fortunately, this outbreak did not signify an emergency^43^ since it was self-limiting once the El Niño event subsided, and the rapid recovery of damselfish and angelfish populations during the subsequent year of La Niña conditions revealed a striking capacity for resilience in this system that warrants further investigation. In addition, the use of fully protected no-take areas, such as those proposed in the new Galapagos Marine Reserve zoning system, may confer resistance to disease spread by limiting cumulative impacts of multiple anthropogenic stressors such as fishing and climate change^4,44^. Even so, ENSO is one of the largest climate oscillations on the planet, and extreme El Niño events are predicted to increase in severity and frequency as global warming progresses^5^. It is possible that future extreme El Niño events will elicit similar outbreaks of disease in wild fish populations in the Galapagos Islands and potentially elsewhere.

## Methods

### Study region

This study took place in the Galapagos Islands, Ecuador. The Galapagos are located 965 km west of the South American mainland, directly on the equator. We quantified sea surface temperature (SST) at 19 study sites in the central Galapagos Islands^24^ between January 13, 2003 and November 21, 2017 using SST values for the entire archipelago from the MODIS satellite dataset at 4 km resolution. We acquired sea surface chlorophyll *a* (in mg/m^3^) as a proxy of primary productivity over the same area, resolution, and time period, also from the MODIS dataset. For both SST and chlorophyll, we calculated the anomaly for each pixel during El Niño by first taking the average value during the sampling month (December 29, 2015 – January 29, 2016; n = 4 weeks), and then subtracting the average for this month across the entire 14-year dataset (n = 679 weeks). This more accurately reflects the strength of the El Niño anomaly than an anomaly constructed against the global average since it takes into account the substantial seasonal and spatial variation in ocean conditions in the Galapagos. We placed the current El Niño event in a historical context using the Oceanic Niño Index (ONI), a metric of the status of the ENSO cycle in the El Niño 3.4 region that is calculated as the anomaly of a 3-month running mean relative to a centered 30-year base period^8^. Positive values signify a warm departure, with El Niño conditions referring to a value above +0.5 °C sustained for at least five consecutive months. Negative values signify a cold departure, with La Niña conditions referring to a value below −0.5 °C sustained for at least five consecutive months.

### Fish surveys

Ulcerative skin disease was first discovered during biannual long-term monitoring by the Witman Lab of fish and benthic communities^24^ at the easternmost island of the Cuatro Hermanos group located to the southeast of Isabela Island on January 8, 2016. Following the initial observation, we conducted surveys to detect the disease and quantify its geographic spread, species distribution, and prevalence rates among populations. Between January 8 and January 29, we characterized the initial outbreak by conducting surveys at 19 sites. At each site, we performed two types of surveys. The first was typical of underwater visual censuses for fish communities: a diver swam along a 10 × 5 m transect and identified all fish to species, estimated their total length, and recorded whether or not each individual showed signs of disease. Our working case definition for ulcerative skin disease was the presence of white lesions, exposed hemorrhagic subcutaneous tissue, and/or scale loss. If disease was detected, the location of lesions on the body and total area of the body affected were also recorded. We carried out 10–20 transects per site between 6 and 15 m depth. This method was implemented in order to assess the total number of affected species and the disease prevalence rates for each species and site.

The second method involved targeted transects searching for disease in the four species in which we observed the highest prevalence rates, “targeted species”: ring-tailed damselfish *Stegastes beebei*, Panamic soldierfish *Myripristis leiognathus*, king angelfish *Holacanthus passer*, and giant damselfish *Microspathodon dorsalis*. For each species, we performed 10–20 consecutive 10 × 5 m band transects between 5 and 20 m depth at each site (see Supplementary Table S2 for transect replication by site and species). This method was implemented in order to obtain rigorous measures of prevalence and general disease characterization for the most susceptible species. To search for diseased fish over a larger reef area (several hundred m^2^), a diver systematically videotaped all reef fish at 6–15 m depths, and we noted any other fish species observed with signs of disease during dives at each site. Both types of censuses were repeated up to 4 times over the course of a 1-month period, and were repeated in March, July, and November 2016, and in January and July 2017. Targeted species transects were not performed at Islote Gardner, La Botella, or Pinzón, so these sites were not included in species-specific analyses. Five transects were performed at Corona del Diablo and Las Palmas. We only included data from the first targeted surveys performed at Cuatro Hermanos East (January 13, 2016), since both population abundance and prevalence decreased across affected species after this point, which was assumed to be the result of disease-associated mortality (Fig. 6).

In order to test for evidence of disease-associated mortality, we used stationary video cameras (GoPro® Hero4) that were deployed as part of the long-term monitoring program. Two cameras were deployed at least 20 m apart and left for one hour to observe fish populations in the absence of diver effects. Videos were then analyzed in 15-minute blocks of time, during which the maximum number of individuals observed in a single frame (“MaxN”) was recorded^45^. We only analyzed data for *S. beebei* and *H. passer*, since these were the only species with high prevalence of disease that are observable using this method, and focused on the Cuatro Hermanos East site where the outbreak was most concentrated. Videos were taken during January and July for the period of 2013–2017, with a total of eight 15-minute segments analyzed per sampling date.

### Pathogen isolation and identification

In order to identify the pathogen responsible for the disease, we collected several individuals of each of the targeted species between January 13 and 23, 2016. We initially sampled 24 fish for histological analysis and molecular comparison to known pathogens. Only one *H. passer* and two *M. dorsalis* were caught due to logistical constraints, while *S. beebei* and *M. leiognathus* were represented by 12 and 9 individuals, respectively. All fish were caught at Cuatro Hermanos East, the site with the highest prevalence rates, except for *M. dorsalis*, which was caught at Caamaño. Fish were caught by “Hawaiian sling” hand spear except for two *S. beebei*, which were caught with a hand net and euthanized via ice slurry immersion. These 24 fish were stored on ice and immediately brought to the lab and dissected, removing a section of affected skin and the liver, heart, kidney, gills, intestine, and spleen. One half of each of these organs was stored in ethanol at 95% concentration for scale-drop disease virus detection based on molecular analyses. The other half was fixated in 10% formalin and subsequently transferred to 70% ethanol for histological analyses.

We subjected tissue samples to a battery of tests to quantify macro- and microscopic pathological disease signs and to identify the causal pathogen at the Roger Williams University Aquatic Diagnostic Laboratory (Bristol, RI), and at the Kennebec River Biosciences laboratory (Richmond, ME). For each of the 24 fish, we performed histological analyses on formalin-fixed tissues from each of the sampled tissue types embedded in paraffin and stained with hematoxylin and eosin.

A separate sample of two individuals of *S. beebei* and one *M. leiognathus* caught by hand spear were kept frozen for viral and bacterial screening. Skin lesions were sampled by swabbing the underside of skin and the surface of underlying muscle tissues in regions at the leading edges of ulcers. Following decontamination of the outer surfaces using 70% isopropanol, kidney and brain tissues were aseptically sampled for bacterial culture using sterile loops and tissue homogenates. Samples from each tissue type were inoculated onto blood agar with 2% NaCl, an in-house marine agar medium (Kennebec River Biosciences, unpublished), *Flexibacter maritimus* agar (FMM)^46^, and Middlebrook 7H11 agar for *Mycobacterium* species^47^. Inoculated media were incubated at 20 °C and 30 °C and monitored for growth over the course of 7 days, or 2 weeks in the case of Mycobacterial medium. Any growth was evaluated and isolates picked for further growth and testing. Following morphological characterization, isolates were identified using a combination of differential stains (Gram, acid-fast), standard biochemical tests (oxidase, catalase, O/F & motility slants), and API-20E test strips (bioMerieux). Results were compared to published data^48^ as well as an in-house database established using API biotypes and matching DNA sequences. We extracted DNA from bacterial colonies using the Qiagen DNeasy kit. Amplification of the 16S rRNA gene region was performed using the 27F/1492R primer set and Platinum SuperFi PCR Mastermix, and 16S rRNA gene sequences were compared with GenBank records.

We also performed viral culture testing in fish cell lines using kidney, spleen, heart, liver, gill, optic nerve and brain tissue samples from the one individual of *M. leiognathus* and two *S. beebei* that were stored and transported frozen. Organ samples were homogenized in 9 parts (weight:volume) Hanks’ balanced salt solution with antibiotics (penicillin & streptomycin), and a 1:10 dilution of the resulting homogenate was prepared in cell culture medium (Leibovitz L-15 with 5% fetal bovine serum). Samples were inoculated within 24 hours in duplicate wells onto 24-well plate cultures of grunt fin (GF-1), grouper (GP), fathead minnow (FHM), epithelioma papulosum cyprini (EPC), spleen of orange spotted grouper (GS)^49^, and in-house barramundi kidney and brain cell lines (Kennebec River Biosciences unpublished) by adsorption for 30 minutes followed by re-feeding with L-15 medium with 5% FBS. Plates prepared in duplicate were incubated at 22 °C and 28 °C. Blind transfers were inoculated after 7–10 days’ incubation and cultures were monitored for a total of 28 days for any observable cytopathic effect.

Cell culture supernatant and ethanol-stored tissues from the initial sample of 24 fish were tested for the presence of scale-drop disease using an agent-specific real time PCR assay for scale-drop disease virus. For this, primer and probe sequences and cycling conditions as described by deGroof *et al*.^50^ were utilized in combination with the Quanta PerfeCTa qPCR ToughMix Low Rox kit according to manufacturer directions. The 20 ul reaction mix consisted of 8.15 ul water, 10 ul 2X buffer, 0.3 ul of each primer (12.5 uM stock), 0.25 ul of probe (10 uM stock) and 250 ng total DNA in 1 ul.

Fish collections and all laboratory methods were approved by the Galapagos National Park, the Ecuadorian Ministry of Environment, and the Institutional Animal Care and Use Committee (IACUC) at Brown University. Fish were collected in accordance with Ecuadorian guidelines and regulations, transported to the United States under procedures established by the United States Fish and Wildlife Service, and processed at Brown University and Kennebec River Biosciences laboratories under a Biological Research Authorization from Brown University.

### Statistical analyses

We examined the relationship between temperature regimes leading up to the disease outbreak and the number of species affected by ulcerative skin disease at each site by performing a linear regression between the number of weeks between September 1, 2015 and January 31, 2016 during which the sea surface temperature remained above 26 °C and the number of species affected with disease (n = 16 sites). Data for the dependent variable (number of species affected) were log (x + 1) transformed to meet regression model assumptions. We also used linear regression to examine the relationship between the mean abundance in monitoring transects of the targeted species and the prevalence of disease across sites (n = 6–16 sites). Arcsine-square root transformation of the dependent variable (prevalence) to meet the assumptions of least-squares regression analysis did not change model fit (Supplementary Table S4), so untransformed values were used. We tested for spatial auto-correlation in disease prevalence across sites using Moran’s I correlation coefficient. Results are shown for *M. leiognathus* and *S. beebei* only, as disease prevalence for the other two species had no significant positive or negative auto-correlation at any distance. Distance classes in km and replication (number of site pairs at that distance class) were as follows: 4.8 (11), 20.3 (10), 31.2 (18), 41.4 (13), 54.0 (26), 66.1 (25), 76.9 (16), 92.3 (12), 97.7 (2), and 112.8 (3). Video-derived abundance data (MaxN^44^) for *H. passer* and *S. beebei* were analyzed using one-way analysis of variance with date as a fixed factor (n = 8 video segments analyzed per species per date, n = 12 sampling dates over 4.5 years). Abundance data for *S. beebei* met assumptions of normality and heteroscedasticity, and and abundance data for *H. passer* met assumptions after a log_10_ (x + 1) transformation.

## Electronic supplementary material

Supplementary Information

Supplementary Video S1

Supplementary Video S2

## Data Availability

All data referenced in this paper and the R code for analyses and figure creation can be found at the following Dryad repository: 10.5061/dryad.57v9d68.
