# Supplementary material for: El Niño drives a widespread ulcerative skin disease outbreak in Galapagos marine fishes
Source: Sci Rep. 2018 Nov 9;8:16602. doi: 10.1038/s41598-018-34929-z (PMC6226461; doi:10.1038/s41598-018-34929-z)
Supplement: Supplementary file 1 — Supplementary Information [file 41598_2018_34929_MOESM1_ESM.pdf]

El Niño drives a widespread ulcerative skin disease outbreak in Galapagos marine fishes

Robert W. Lamb*, Franz Smith, Anaide W. Aued, Pelayo Salinas de León, Jenifer Suarez, Marta Gomez-Chiari, Roxanna Smolowitz, Cem Giray, and Jon D. Witman

Supplementary Information

***Corresponding author email: robert_lamb@brown.edu**

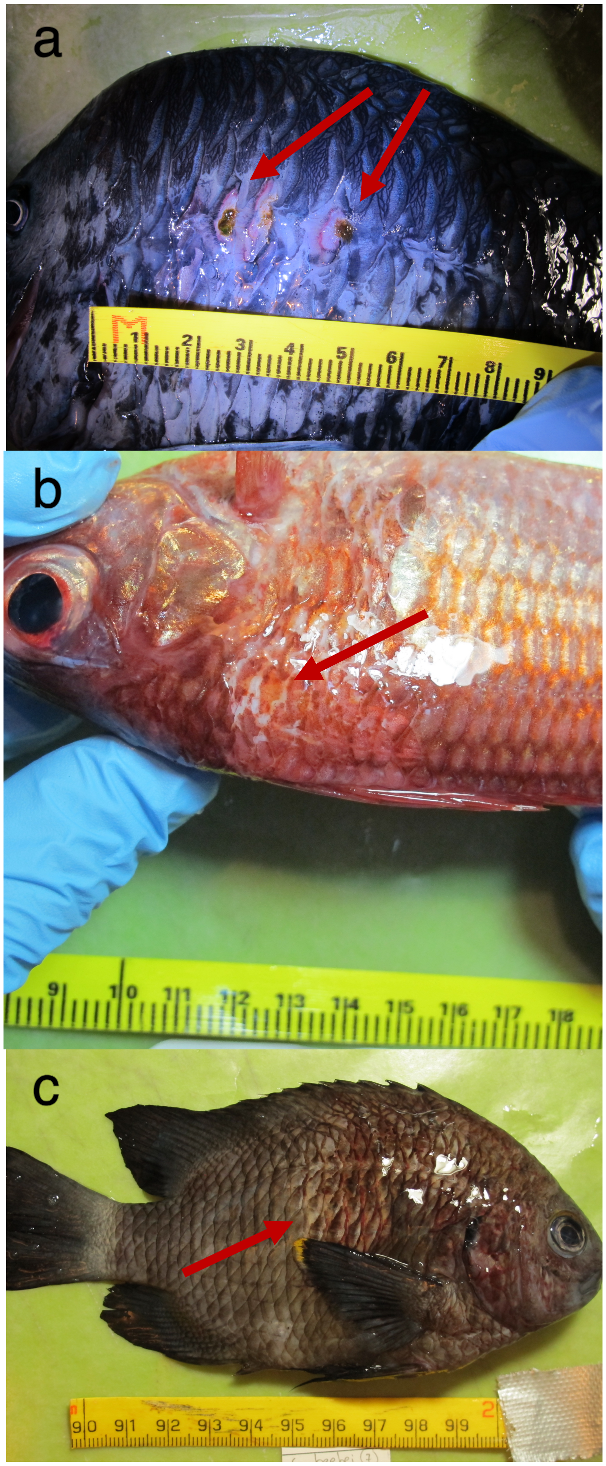

Supplementary Figure S1 | Macroscopic manifestations of ulcerative skin disease. (a) Ulcers below the dorsal fin of a *Microspathodon dorsalis* taken from Camaño. (**b)** Scale loss on the dorsal side of a *Myripristis leiognathus* taken from Cuatro Hermanos (East). (**c)** Extensive scale

loss and hemorrhage along the entire anterior portion of a *Stegastes beebei* taken from Cuatro Hermanos (East).

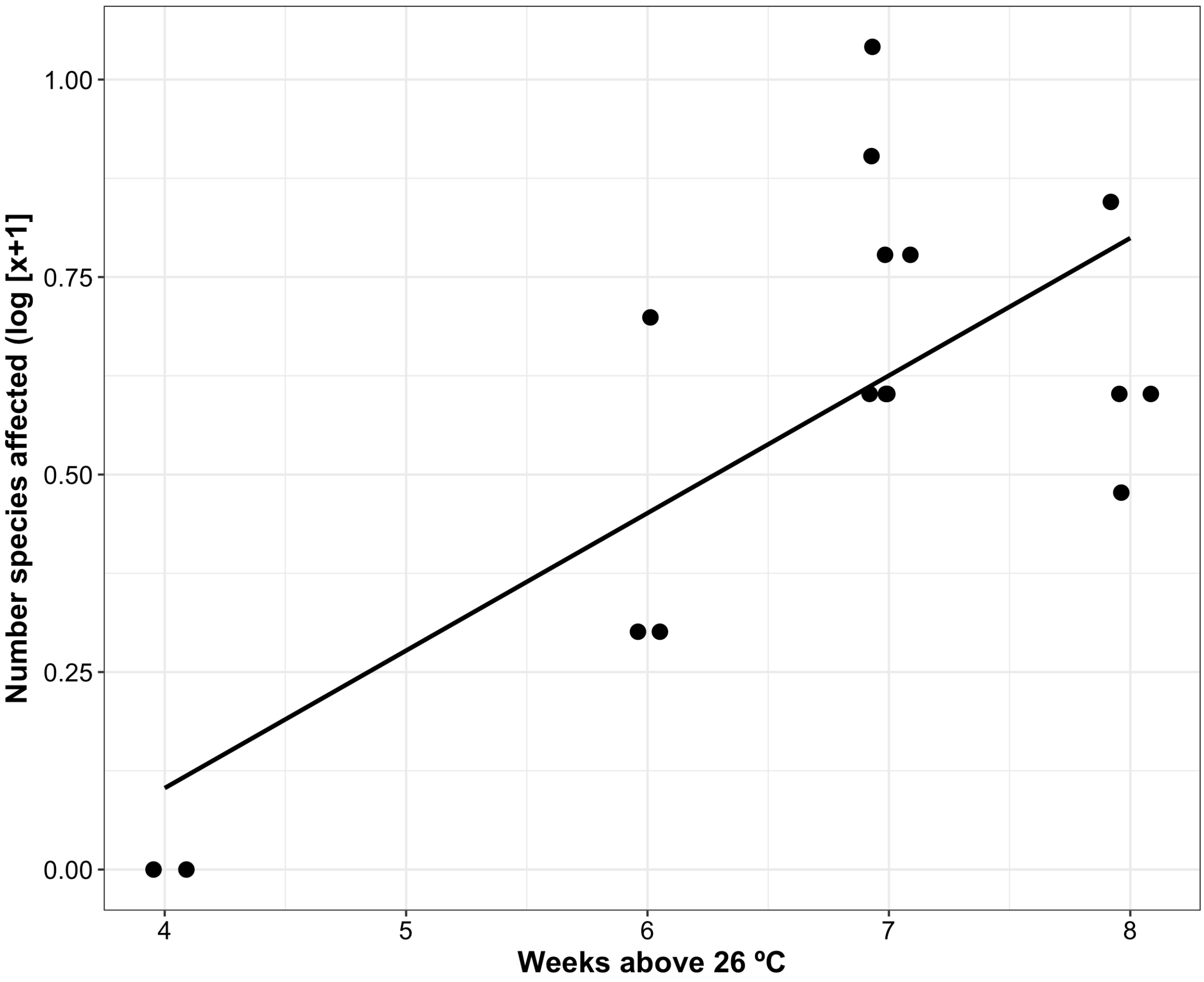

Supplementary Figure S2 | Relationship between temperature and number of species affected by ulcerative skin disease across sites. Points are survey sites (*n* = 16). The X-axis represents the number of weeks between Sept. 1, 2015 and Jan 31, 2016, during which mean sea surface temperature surpassed 26 °C. The y-axis represents the number of species observed with ulcerative skin disease (log[x+1]-transformed to meet linear regression model assumptions). A very slight jitter was added to point locations on the x-axis to visualize overlapping points, but the regression line represents true values. There was a significant positive linear relationship between the two variables ($y = 0.174x – 0.593$, $P = 0.0012$, $R^2 = 0.505$).

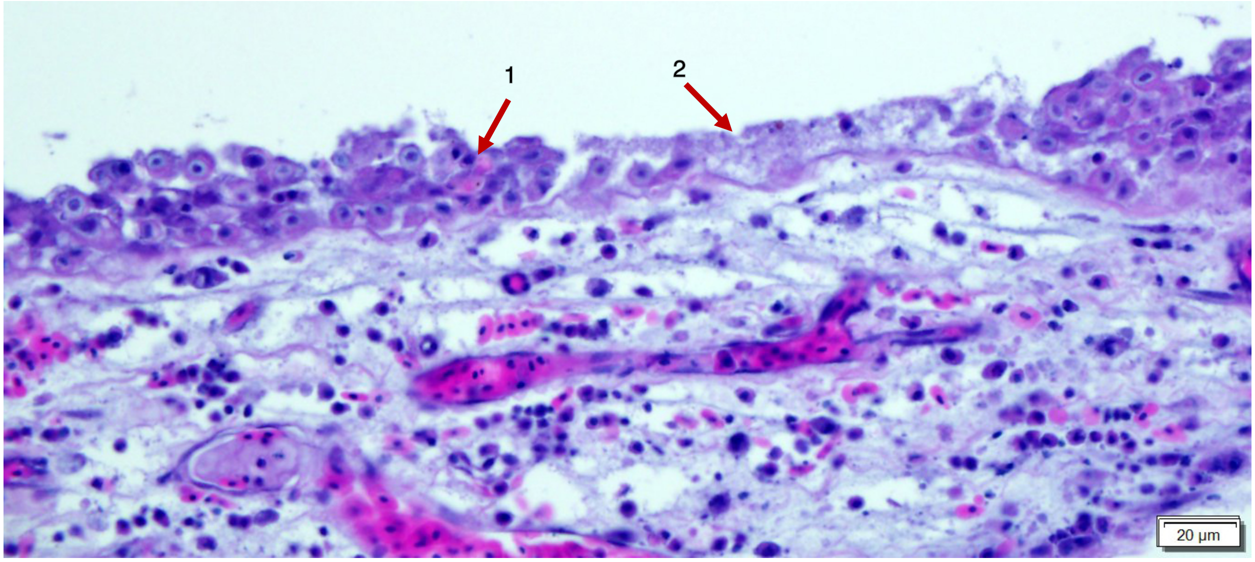

Supplementary Figure 3 | Microscopic histology slide of ulcerative skin disease. Lesions characterized by diffuse moderate macrophagic and lymphocytic dermatitis with vascular congestion, mild dermal hemorrhage and severe diffuse edema. Arrows indicate vacuolation of epithelial nuclei and rare cytoplasmic eosinophilic inclusions (**1**) and edema of epidermal cells with ulceration associated with debris and possible bacteria (**2**) (6 µm sections of paraffin embedded tissues stained with hematoxylin and eosin).

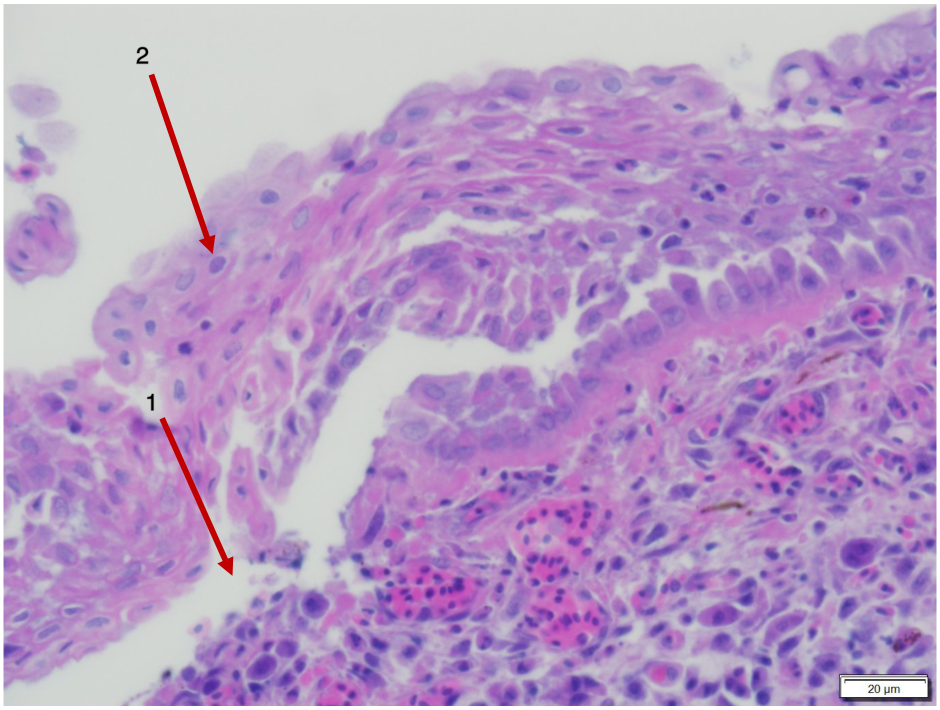

Supplementary Figure 4 | Microscopic histology slide of epithelial degloving. All layers of the squamous stratified epithelium are dead or edematous resulting in separation of most of the layers of the epithelium (degloving) from the underlying basal epithelial cells resulting in erosion (**1**) and ulceration. Severe macrophagic and lymphocytic infiltration with vascular congestions and mild hemorrhage is noted in the underlying dermis. Amphiphilic to eosinophilic inclusions are rarely noted in the nuclei (**2**) (6 µm sections of paraffin-embedded tissues stained with hematoxylin and eosin).

Supplementary Table S1: Extended literature review of reports of ulcerative skin disease in fish populations. We tabulated reports of ulcerative skin disease in wild populations as well as selected cases of similar symptoms to those in our study reported from aquaculture. Here we summarized the species affected, associated pathogens, disease signs, and environmental triggers.

Author	Date	Publication	Citation	Primary species	Number species affected	Pathogen	Disease signs	Environmental context	Notes
Noga et al	1996	Marine Pollution Bulletin	Vol 32, No.2, pp. 219-224	Striped bass *Morone saxiatilis*	2	Dinamoebales (dinoflagellate)	Diffuse erythema, dull luster of skin, loss of epithelium extendeing to basement membrane, myonecrosis, ulcers with gram-negative bacterial infection (*Aeromonas*), intra and extra-cellular edema, necrosis.	Dinoflagellate blooms; esturaine eutrophication	Noga et al 1996 performed laboratory trials with a novel dinoflagellate (new family, order Dinamoebales) that was causing fish kills in Atlantic estuaries. Yielded 100% mortality within 24-48 hrs in striped bass and tilapia. Histology of non-skin tissues was normal. Believed triggers are coastal eutrophication and immunosuppression. Ulcers occur rapidly and are colonized by mulitple environmental pathogens.
McKenzie and Hall	1976	Australian Veterinary Journal	Vol 250, No. 5, pp. 230-231	Mullet *(Mugil cephalus)*	1	Aphanomyces	Patches of dull skin lead to scale loss and ulcers, erythematous dermatitis; may involve the eye. Extravasation of leucocytes forming perivascular cuffs	Drops in temperature, salinity, DO, pH	Bundaberg disease or Red Spot Disease (RSD) was first observed in mullet (*Mugil* spp.) in Australia
Noga	1993	Pathobiology of marine and estuarine organisms	Fungal diseases of marine and estuarine fishes, pp. 85-100	Atlantic menhaden (*Brevoortia tyrannus*), Barramuni (*Lates calcifer*), goldfish, catfish, bluegill	>30 reported, but different outbreaks and suspected pathogens	Several fungal pathogens: *Aphanomyces, Achlya, Saprolegnia* common	Surface lesions progress to deep ulcers penetrating supepidermal muscle. Large necrotic pits result, with masses of hyphae, bacteria, and necrotized muscle that can slough off, leaving a cavity in the skin.	Ulcerative mycosis is common in estuarine and aquaculture fishes and is believed to be triggered by low salinity and high nutrient loads from coastal runoff.	Noga 1993 reviews fungal diseases affecting marine fish populations. Points out ulcerative mycosis is generally associated with large outbreaks in atlantic menhaden off the US Atlantic coast, may be picked up from sediments when they dive down to feed. Occurrences from New England to Florida
Boonyaratpalin	1985	FAO special report	Fish disease outbreak in Burma; report number TCP/BUR/4402	*Ophicephalus* spp.	1	Aphanomyces	Surface lesions and ulcerated skin	Cooling temperatures	First mention of Epizootic Ulcerative Syndrome; later synonomized with Red Spot Disease (Callinan et al 1995)
Callinan et al	1995	Diseases of aquatic organisms	Vol 21, pp 233-238	Several species of *Mugil, Arius,* and *Scatophagus*	>15	Aphanomyces	Surface lesions, hyphal infiltration	NA	Pathological study found the same fungal pathogen *Aphanomyces* responsible for both Red Spot Disease (RSD)/Bundaberg disease and Epizootic Ulcerative Syndrome (EUS) across Australia and Philippines
Lunder et al	1995	Diseases of aquatic organisms	Vol 23, pp 39 – 49.	Salmo salar (atlantic salmon)	1	Vibrio bacteria	Ulcers over scale-covered skin, internal hemorrhage and	Crowding in aquaculture, cold temperatures	Winter ulcer appears on farmed atlantic salmon in cold temperatures, observed in Iceland,

						petechia	("winter ulcer")	Norway, and Scotland
Lunder et al	2000	International Journal of Systematic and Evolutionary Microbiology	Vol 50, 427 – 450.	Salmo salar (atlantic salmon)	1	Vibrio viscosus, V. wodanis	Ulcers over scale-covered skin, internal hemorrhage and petechia	Crowding in aquaculture, cold temperatures ("winter ulcer")	DNA analysis confirmed Vibrio cause of winter ulcer
Toranzo et al	1991	Fish Pathology	Vol 26, 55 – 60	Salmo salar (atlantic salmon)	1	Aeromonas salmonicida	Liquefactive necrosis, scale loss, deep ulcers, hemorrhagic septicemia	Crowding in aquaculture	Common disease in farmed atlantic salmon
Magariños et al	1995	Diseases of aquatic organisms	Vol 21, 103 – 108	Seabream, sole, turbot, salmonids, seabass, flounder (cultured fishes)	8	Tenacibaculum maritimum	Eroded and hemorrhagic mouth, fin rot, ulcerative skin loss, and involvement of internal organs	High temperatures (above 15 C), concomittant environmental stress (skin lesions, crowding,	Tested skin entry pathways for T. maritimum
Lewis and Chinabut	1999	Mycobacteriosis and Nocardiosis. In: Woo, P.T.K., Bruno, D.W. (Eds.), Fish Diseases and Disorders	Vol 3, pp 319 - 340	Cod, halibut, striped bass, atlantic mackerel (all wild)	200	Mycobacterium marinum	Tubercle granulomas in the spleen, kdney, and liver, scale loss and hemorrhagic lesions penetrating to the musculature in severe cases	Common in a variety of situations including aquaculture, aquaria, and wild populations	Affects over 200 known species, 4 noted as wild populations. This is a chronic disease that remains asymptomatic for long periods
Love et al	1981	Science	Vol. 214, Issue 4525, pp. 1139-1140	Chromis punctipinnis	3	Vibrio damsela	Skin ulcers	NA	Laboratory tests confirmed causality in the blacksmith (*C. punctipinnis*) and other damselfish. Also observed in sandbar and spiny dogfish sharks (see Grimes et al 1984)
Larsen et al	1978	Veterinary Science Communications	Vol 2, 207-216.	Cod *(Gadus morhua*)	1	Vibrio anguillarum	Skin ulcers	Coastal pollution including town effluence, sugar and cellulose plant outflows	Ulcer disease common in polluted waters, absent from other areas. V. anguillarum readily infected pre-existing ulcers and induced systemic vibriosis but did not cause ulcers to occur de novo
Gibson-Kueh et al	2012	Journal of Fish Diseases	Vol 35, 19-27	Lates calcarifer (barramundi)	1	Iridovirus	Skin ulcers, scale loss	Crowding in aquaculture	Ulcers and scale loss usually over the posterior half of the body, was found to be caused by a novel iridovirus
de Groof et al	2015	PloS One	https://doi.org/10.1371/journal.ppat.1005074	Lates calcarifer (barramundi)	1	Iridovirus	Skin ulcers, scale loss	Crowding in aquaculture	Ulcers and scale loss usually over the posterior half of the body, was found to be caused by a novel iridovirus
Mahoney et al	1973	Transactions of the American Fisheries Society	Vol 102, 596-605	Bluefish, flounder, weakfish	22	Aeromonas, Pseudomonas, Vibrio	Skin ulcers, fin rot, blindness	Intense coastal pollution including sewage discharge and industrial waste	Many species of wild fish caught in survey trawls in the New York Bight had similar symptoms of fin rot and scale loss with skin ulcers, attributed to massive quantities of bacteria blooming in coastal pollution
Hansen et al	1990	Journal of Fish Diseases	Vol 13: 93–96	Mahi-mahi *(Coryphaena hippurus)*	1	*Pantoea agglomerans*	Hemorrhagic disease, ulcers of the skin, eyes, and musculature	Cold water, poor nutrition, crowding in aquaculture	P. agglomerans was isolated from lesions caused by aquaculture crowding and cold water in Mahi-mahi
Wellings et al	1977	Annals of the New York	298(1), 290-304	Rock sole *(Lepidopsetta*	1	Unknown	Epidermal papilloma	Natural incidence reported from trawl surveys	Surveyed wild fish disease prevalence and observed epidermal papilloma (<1%

	Academy of Sciences	*bilineata*)		(non-epidemic)	incidence) in rock sole (Lepidopsetta bilinear), lymphocytosis (<2%) in yellowfin sole (Limanda aspera), and pseudobranchial tumors (<8%) in cod (Gadus macrocephalus)

Supplementary Table S2: List of study sites. A total of 19 sites were surveyed during the ulcerating skin disease outbreak in January 2018. The site abbreviation code (referenced in Figure 3), geographic position, number of focal species fish transects, total richness and percent of species observed with disease.

Site	Code	Easting	Northing	*n*	Richness	% spp infected
Baltra*	BAL	803393	9954459	20	63	4.8
Camaño Exposed	CAM	802822.7	9915869	10	30	16.7
CH (East)*	CHE	750618.3	9906145	20	47	21.3
CH (Gemelo East)	CGE	745312	9906116	11	30	10.0
CH (Gemelo West)	CGW	743443.4	9906369	20	31	22.6
CH (Grande)	CHG	747955.6	9904560	20	21	4.8
Champion*	CHA	791020.6	9863149	20	60	5.0
Corona del Diablo	CD	786811.2	9865479	5	32	6.3
Daphne Menor*	DM	794620.1	9956334	20	38	5.3
Guy Fawkes*	GF	776904.6	9944797	20	55	5.5
Islote Gardner*	GAR	801109.7	9852964	NA	38	0.0
Islote Tortuga	IT	735928.2	9888406	10	25	16.0
La Botella*	LB	778317.8	9857288	NA	49	0.0
Las Cuevas*	LC	794198.9	9860020	20	57	1.8
Las Palmas Chicas	PAL	773339.8	9924915	5	23	0.0
Pinzón*	PIN	757552.3	9934638	NA	45	0.0
Roca Cousins*	RC	770133.1	9973785	20	55	10.9

Rocas Beagle*	RB	764013.8	9954343	20	54	5.6
Rocas Gordon*	GOR	818291.7	9937366	10	36	13.9

CH: Cuatro Hermanos

*Sites that have been monitored bi-annually since at least 2002

Supplementary Table S3: Statistical tests of differences in population size among years for *Holacanthus passer* and *Stegastes beebei* at Cuatro Hermanos East. We performed ANOVA and multiple comparisons tests on abundance for *H. passer* and *S. beebei* population size over the study period of January 2013 – July 2017 at the site of Cuatro Hermanos East, where the disease outbreak reached its highest prevalence. All data are in MaxN, the maximum number of individuals observed together in a single frame over a 10-minute period of video analyzed. MaxN for *H. passer* is standardized to the depth of view of the water column during the video analyzed (visibility, in meters), and log-transformed to meet assumptions of normality and homogeneity of variance. MaxN for *S. beebei* is standardized to the benthic area visible in the video (m^2), and is untransformed.

One-way ANOVA
Factor: Date
Response variable: Abundance (MaxN) *Holacanthus passer*

ANOVA table

	Df	Sum Sq	Mean Sq	F value	Pr(>F)
Date	11	42.441	3.8583	9.7381	<.001
Residuals	81	32.093	0.3962		

Tukey multiple comparison of means

Pairwise comparison (year)	Mean difference	Lower 95% CI	Upper 95% CI	Adjusted p-value
2013-08-09-2013-02-19	-0.043181799	-1.1023886	1.01602499	1
2014-01-10-2013-02-19	-0.590777696	-1.6499845	0.46842909	0.769433
2014-08-02-2013-02-19	-0.201530096	-1.3456042	0.94254399	0.9999821
2015-01-08-2013-02-19	0.096682282	-0.9625245	1.15588907	1
2015-07-14-2013-02-19	-0.048476922	-1.1076837	1.01072986	1
2016-01-08-2013-02-19	-1.311987459	-2.3711942	-0.25278067	0.0041241
2016-03-25-2013-02-19	-1.909683216	-3.0060664	-0.81330003	0.000006

2016-08-04-2013-02-19	-1.462871214	-2.522078	-0.40366443	0.000747
2016-11-16-2013-02-19	0.023315929	-1.0358909	1.08252271	1
2017-01-12-2013-02-19	0.275466928	-0.7837399	1.33467371	0.9991924
2017-07-19-2013-02-19	-0.635845992	-1.6950528	0.42336079	0.6784909
2014-01-10-2013-08-09	-0.547595897	-1.6068027	0.51161089	0.8441051
2014-08-02-2013-08-09	-0.158348298	-1.3024224	0.98572579	0.9999985
2015-01-08-2013-08-09	0.139864081	-0.9193427	1.19907087	0.9999991
2015-07-14-2013-08-09	-0.005295123	-1.0645019	1.05391166	1
2016-01-08-2013-08-09	-1.26880566	-2.3280124	-0.20959888	0.0065461
2016-03-25-2013-08-09	-1.866501417	-2.9628846	-0.77011823	0.0000105
2016-08-04-2013-08-09	-1.419689416	-2.4788962	-0.36048263	0.0012351
2016-11-16-2013-08-09	0.066497728	-0.9927091	1.12570451	1
2017-01-12-2013-08-09	0.318648726	-0.7405581	1.37785551	0.9969959
2017-07-19-2013-08-09	-0.592664194	-1.651871	0.46654259	0.7658588
2014-08-02-2014-01-10	0.389247599	-0.7548265	1.53332168	0.9914705
2015-01-08-2014-01-10	0.687459978	-0.3717468	1.74666676	0.5654752
2015-07-14-2014-01-10	0.542300774	-0.516906	1.60150756	0.8522284
2016-01-08-2014-01-10	-0.721209763	-1.7804165	0.33799702	0.4909299
2016-03-25-2014-01-10	-1.31890552	-2.4152887	-0.22252234	0.0061845
2016-08-04-2014-01-10	-0.872093519	-1.9313003	0.18711327	0.2123894
2016-11-16-2014-01-10	0.614093625	-0.4451132	1.67330041	0.723717
2017-01-12-2014-01-10	0.866244623	-0.1929622	1.92545141	0.220704
2017-07-19-2014-01-10	-0.045068297	-1.1042751	1.01413849	1
2015-01-08-2014-08-02	0.298212379	-0.8458617	1.44228646	0.9991753
2015-07-14-2014-08-02	0.153053174	-0.9910209	1.29712726	0.999999
2016-01-08-2014-08-02	-1.110457363	-2.2545314	0.03361672	0.0653453
2016-03-25-2014-08-02	-1.708153119	-2.8867296	-0.52957661	0.0003149
2016-08-04-2014-08-02	-1.261341118	-2.4054152	-0.11726703	0.0182745
2016-11-16-2014-08-02	0.224846026	-0.9192281	1.36892011	0.999946
2017-01-12-2014-08-02	0.476997024	-0.6670771	1.62107111	0.9596456
2017-07-19-2014-08-02	-0.434315896	-1.57839	0.70975819	0.979705
2015-07-14-2015-01-08	-0.145159204	-1.204366	0.91404758	0.9999987
2016-01-08-2015-01-08	-1.408669741	-2.4678765	-0.34946296	0.0014018
2016-03-25-2015-01-08	-2.006365498	-3.1027487	-0.90998231	0.0000017
2016-08-04-2015-01-08	-1.559553497	-2.6187603	-0.50034671	0.0002339
2016-11-16-2015-01-08	-0.073366353	-1.1325731	0.98584043	1
2017-01-12-2015-01-08	0.178784646	-0.8804221	1.23799143	0.9999884
2017-07-19-2015-01-08	-0.732528275	-1.7917351	0.32667851	0.4663747
2016-01-08-2015-07-14	-1.263510537	-2.3227173	-0.20430375	0.0069214
2016-03-25-2015-07-14	-1.861206294	-2.9575895	-0.76482311	0.0000112

2016-08-04-2015-07-14	-1.414394293	-2.4736011	-0.35518751	0.0013127
2016-11-16-2015-07-14	0.071792851	-0.9874139	1.13099964	1
2017-01-12-2015-07-14	0.32394385	-0.7352629	1.38315063	0.996532
2017-07-19-2015-07-14	-0.58736907	-1.6465759	0.47183771	0.7758308
2016-03-25-2016-01-08	-0.597695757	-1.6940789	0.49868743	0.7940263
2016-08-04-2016-01-08	-0.150883755	-1.2100905	0.90832303	0.999998
2016-11-16-2016-01-08	1.335303388	0.2760966	2.39451017	0.0031965
2017-01-12-2016-01-08	1.587454387	0.5282476	2.64666117	0.0001659
2017-07-19-2016-01-08	0.676141467	-0.3830653	1.73534825	0.5906038
2016-08-04-2016-03-25	0.446812001	-0.6495712	1.54319518	0.9656857
2016-11-16-2016-03-25	1.932999145	0.836616	3.02938233	0.0000045
2017-01-12-2016-03-25	2.185150143	1.088767	3.28153333	0.0000002
2017-07-19-2016-03-25	1.273837223	0.177454	2.37022041	0.0097375
2016-11-16-2016-08-04	1.486187144	0.4269804	2.54539393	0.000567
2017-01-12-2016-08-04	1.738338142	0.6791314	2.79754493	0.0000245
2017-07-19-2016-08-04	0.827025222	-0.2321816	1.88623201	0.2821603
2017-01-12-2016-11-16	0.252150999	-0.8070558	1.31135778	0.9996486
2017-07-19-2016-11-16	-0.659161922	-1.7183687	0.40004486	0.6280729
2017-07-19-2017-01-12	-0.91131292	-1.9705197	0.14789386	0.1623097

One-way ANOVA
Factor: Date
Response variable: Abundance (MaxN) *Stegastes beebei*

ANOVA table

	Df	Sum Sq	Mean Sq	F-value	Pr(>F)
Date	11	1535.4	139.578	8.396	<.001
Residuals	81	1346.6	16.624		

Tukey multiple comparison of means

Pairwise comparison (year)	Mean difference	Lower 95% CI	Upper 95% CI	Adjusted p-value
2013-08-09-2013-02-19	1.40277778	-5.45827223	8.26382779	0.99992
2014-01-10-2013-02-19	-3.65972222	-10.52077223	3.20132779	0.8157732
2014-08-02-2013-02-19	-4.68055556	-12.09133656	2.73022545	0.6064526
2015-01-08-2013-02-19	-1.38888889	-8.2499389	5.47216112	0.9999275
2015-07-14-2013-02-19	0.95486111	-5.9061889	7.81591112	0.9999984
2016-01-08-2013-02-19	-5.51388889	-12.3749389	1.34716112	0.2430601

2016-03-25-2013-02-19	-9.3452381	-16.44709957	-2.24337662	0.0016612
2016-08-04-2013-02-19	-11.88888889	-18.7499389	-5.02783888	0.0000068
2016-11-16-2013-02-19	1.31944444	-5.54160557	8.18049446	0.9999566
2017-01-12-2013-02-19	-5.13888889	-11.9999389	1.72216112	0.3425686
2017-07-19-2013-02-19	-3.88194444	-10.74299446	2.97910557	0.7531164
2014-01-10-2013-08-09	-5.0625	-11.92355001	1.79855001	0.3652412
2014-08-02-2013-08-09	-6.08333333	-13.49411434	1.32744767	0.2160807
2015-01-08-2013-08-09	-2.79166667	-9.65271668	4.06938334	0.9660752
2015-07-14-2013-08-09	-0.44791667	-7.30896668	6.41313334	1
2016-01-08-2013-08-09	-6.91666667	-13.77771668	-0.05561666	0.0463611
2016-03-25-2013-08-09	-10.74801587	-17.84987734	-3.6461544	0.0001368
2016-08-04-2013-08-09	-13.29166667	-20.15271668	-6.43061666	0.0000004
2016-11-16-2013-08-09	-0.08333333	-6.94438334	6.77771668	1
2017-01-12-2013-08-09	-6.54166667	-13.40271668	0.31938334	0.0760509
2017-07-19-2013-08-09	-5.28472222	-12.14577223	1.57632779	0.3014449
2014-08-02-2014-01-10	-1.02083333	-8.43161434	6.38994767	0.9999986
2015-01-08-2014-01-10	2.27083333	-4.59021668	9.13188334	0.9932087
2015-07-14-2014-01-10	4.61458333	-2.24646668	11.47563334	0.5102482
2016-01-08-2014-01-10	-1.85416667	-8.71521668	5.00688334	0.9988499
2016-03-25-2014-01-10	-5.68551587	-12.78737734	1.4163456	0.2481176
2016-08-04-2014-01-10	-8.22916667	-15.09021668	-1.36811666	0.0064358
2016-11-16-2014-01-10	4.97916667	-1.88188334	11.84021668	0.3907855
2017-01-12-2014-01-10	-1.47916667	-8.34021668	5.38188334	0.9998652
2017-07-19-2014-01-10	-0.22222222	-7.08327223	6.63882779	1
2015-01-08-2014-08-02	3.29166667	-4.11911434	10.70244767	0.9378213
2015-07-14-2014-08-02	5.63541667	-1.77536434	13.04619767	0.3200686
2016-01-08-2014-08-02	-0.83333333	-8.24411434	6.57744767	0.9999998
2016-03-25-2014-08-02	-4.66468254	-12.29895423	2.96958915	0.654147
2016-08-04-2014-08-02	-7.20833333	-14.61911434	0.20244767	0.0641483
2016-11-16-2014-08-02	6	-1.41078101	13.41078101	0.2334488
2017-01-12-2014-08-02	-0.45833333	-7.86911434	6.95244767	1
2017-07-19-2014-08-02	0.79861111	-6.6121699	8.20939212	0.9999999
2015-07-14-2015-01-08	2.34375	-4.51730001	9.20480001	0.9911846
2016-01-08-2015-01-08	-4.125	-10.98605001	2.73605001	0.6764271
2016-03-25-2015-01-08	-7.95634921	-15.05821068	-0.85448774	0.0151785
2016-08-04-2015-01-08	-10.5	-17.36105001	-3.63894999	0.0001093
2016-11-16-2015-01-08	2.70833333	-4.15271668	9.56938334	0.9727999
2017-01-12-2015-01-08	-3.75	-10.61105001	3.11105001	0.7913351
2017-07-19-2015-01-08	-2.49305556	-9.35410557	4.36799446	0.985535
2016-01-08-2015-07-14	-6.46875	-13.32980001	0.39230001	0.0833919

2016-03-25-2015-07-14	-10.30009921	-17.40196068	-3.19823774	0.0003109
2016-08-04-2015-07-14	-12.84375	-19.70480001	-5.98269999	0.0000009
2016-11-16-2015-07-14	0.36458333	-6.49646668	7.22563334	1
2017-01-12-2015-07-14	-6.09375	-12.95480001	0.76730001	0.1309792
2017-07-19-2015-07-14	-4.83680556	-11.69785557	2.02424446	0.4361496
2016-03-25-2016-01-08	-3.83134921	-10.93321068	3.27051226	0.8046654
2016-08-04-2016-01-08	-6.375	-13.23605001	0.48605001	0.0936896
2016-11-16-2016-01-08	6.83333333	-0.02771668	13.69438334	0.0519045
2017-01-12-2016-01-08	0.375	-6.48605001	7.23605001	1
2017-07-19-2016-01-08	1.63194444	-5.22910557	8.49299446	0.9996514
2016-08-04-2016-03-25	-2.54365079	-9.64551226	4.55821068	0.9870901
2016-11-16-2016-03-25	10.66468254	3.56282107	17.76654401	0.0001596
2017-01-12-2016-03-25	4.20634921	-2.89551226	11.30821068	0.6963935
2017-07-19-2016-03-25	5.46329365	-1.63856782	12.56515512	0.3032633
2016-11-16-2016-08-04	13.20833333	6.34728332	20.06938334	0.0000004
2017-01-12-2016-08-04	6.75	-0.11105001	13.61105001	0.0580142
2017-07-19-2016-08-04	8.00694444	1.14589443	14.86799446	0.0092053
2017-01-12-2016-11-16	-6.45833333	-13.31938334	0.40271668	0.0844874
2017-07-19-2016-11-16	-5.20138889	-12.0624389	1.65966112	0.3245851
2017-07-19-2017-01-12	1.25694444	-5.60410557	8.11799446	0.9999733

Supplementary Table S4: Statistical test of density dependence of disease prevalence using arcsin-square root-transformed data. We tested for improved fit of data to a linear model after arcsin-square root-transforming prevalence values as predicted by population density for each of the four primary species of fish affected by ulcerative skin disease. No change in model fit was observed in model outputs for any of the four species.

Holacanthus passer

	Estimate	Std. Error	t-value	p-value
(Intercept)	0.0058	0.0047	1.2310	0.2419
mean_abund	0.0017	0.0008	2.1170	0.0558

Residual standard error: 0.01251 on 12 DF
Adjusted r-squared: 0.2113
F-statistic: 4.482

Stegastes beebei

	Estimate	Std. Error	t-value	p-value
(Intercept)	-0.0024	0.0058	-0.4090	0.6886
mean_abund	0.0013	0.0003	4.7050	0.0003

Residual standard error: 0.01396 on 15 DF
Adjusted r-squared: 0.5691
F-statistic: 22.13

Myripristis leiognathus

	Estimate	Std. Error	t-value	p-value
(Intercept)	0.0048	0.0055	0.8620	0.4057
mean_abund	0.0036	0.0012	2.9620	0.0119

Residual standard error: 0.01509 on 12 DF
Adjusted r-squared: 0.3742

F-statistic: 8.774

Microspathodon dorsalis

	Estimate	Std. Error	t-value	p-value
(Intercept)	0.0120	0.0122	0.9860	0.3620
mean_abund	0.0014	0.0023	0.6140	0.5620

Residual standard error: 0.02791 on 6 DF
Adjusted r-squared: -0.09765
F-statistic: 0.3773

Supplementary Video S1: Video compilation of disease signs in *H. passer, M. leiognathus,* and *S. beebei* at Cuatro Hermanos East.

Supplementary Video S2: Video of abnormal behavior expressed by *M leiognathus* with signs of ulcerative skin disease soliciting cleaning by the cleaner fish *Johnrandallia nigrirostris*.

Supplementary Text S1: All R custom code used for data analysis and figure creation. Note: for initial submission only; final code file will be included with data in Dryad repository upon acceptance.
